# The physician-scientists: rare species in Africa

**DOI:** 10.11604/pamj.2018.29.8.13239

**Published:** 2018-01-04

**Authors:** Anthonio Oladele Adefuye, Henry Ademola Adeola, Johan Bezuidenhout

**Affiliations:** 1 Division Health Sciences Education, Office of the Dean, Faculty of Health Sciences, University of the Free State, Bloemfontein, South Africa; 2 Department of Oral and Maxillofacial Pathology, Faculty of Dentistry, University of the Western Cape and Tygerberg Hospital, Cape Town, South Africa; 3 Division of Dermatology, Department of Medicine, Faculty of Health Sciences and Groote Schuur Hospital, University of Cape Town, Cape Town, South Africa

**Keywords:** Physician-scientist, biomedical research, Africa

## Abstract

There is paucity of physician-scientists in Africa, resulting in overt dependence of clinical practice on research findings from advanced “first world” countries. Physician-scientists include individuals with a medical degree alone or combined with other advanced degrees (e.g. MD/MBChB and PhD) with a career path in biomedical/ translational and patient-oriented/evaluative science research. The paucity of clinically trained research scientists in Africa could result in dire consequences as exemplified in the recent Ebola virus epidemic in West Africa, where shortage of skilled clinical scientists, played a major role in disease progression and mortality. Here we contextualise the role of physician-scientist in health care management, highlight factors limiting the training of physician-scientist in Africa and proffer implementable recommendations to address these factors.

## Introduction

While, countries in Africa bear the highest burden of poverty-related and neglected infectious diseases worldwide, health research originating from these regions remains very low [1]. The high incidence of transmittable and communicable disease such as malaria (88% of 214 million new cases of malaria worldwide in 2015) [2], HIV/AIDS and Tuberculosis [3] and non-communicable diseases including cardiovascular disease and tobacco related conditions [4] necessitates the need for strengthening clinical research in Africa. Although, the problems facing medical research in Africa is multifaceted, a major identifiable problem is the lack of proper career structures within medical schools or biomedical research institutions in Africa [5]. The curricula for biomedical science courses in many universities in Africa do not inspire students to consider a career in medical research and lacks teaching on recent advances in the field of medicine [5]. As the 21^st^ century unfolds, unprecedented discovery in the life sciences has redefined ways in which medical research is being conducted and, ultimately, how biomedical research leads to improvements in health [6]. However, while research scientists continually produce new knowledge relevant to clinical care, a good majority of these are not incorporated into clinical practice [7] and clinical problems are rarely translated into research projects [8]. Physician-scientists play a vital role in filling this gap as they engage in both clinical practice and research [9]. Physician-scientists include those with a medical degree (MD or MBChB) alone or combined with other advanced degrees (i.e. MD/MBChB and PhD) with a career path in research anywhere along the entire spectrum of biomedicine, ranging from basic science, through translational and patient-oriented research, to the evaluative sciences [10]. This enables them to translate research findings into clinic practice as well as develop clinically relevant research questions based on clinical issues they encounter in practice or at bedside [11,12]. In Africa, there is paucity of these group of heterogeneous individuals (Physician-scientists) [13, 14].

## Commentary

**Paucity of physician-scientist workforce in Africa: lesson from the Ebola virus outbreak**: In the ever changing clinical practice environment of the 21^st^ century medicine [15], biomedical research has been acknowledged as a powerful tool for solving health challenges [16] and plays a very important role in the provision of healthcare [17]. However, the act of engaging in biomedical research is lacking in the overwhelming majority of countries in Africa [16]. Although, there has been an improvement in health research publications (from 3623 in 2000 to 12,709 in 2014) [18], 52% of which was contributed by three countries (South Africa, Nigeria and Kenya) [18], practice of biomedical research in the continent is yet to reach an acceptable level [17]. Gross dependence of medical practice on research findings from the technologically advanced world is still the norm in most African countries and has presented with numerous challenges in the management of some diseases [17]. In order to tackle the present scourge of enormous healthcare burdens appropriately, countries in Africa need well-trained physician-scientists workforce to lead research endeavours and to train future clinical researcher [14]. In a continent plagued by massive and continuous emigration of physicians to high income countries [19], little is known about the present capacity of the physician-scientists workforce in Africa. The paucity of clinically trained research scientists in Africa could present with dire consequences as exemplified in the recent Ebola virus epidemic in West Africa. The recent Ebola virus epidemic that claimed the life of 11, 310 individuals in three countries in West Africa (Guinea, Liberia and Sierra Leone) [20] exposed the weakness and fragility of many of Africa's healthcare systems [21]. One of the major challenges of combating the Ebola virus epidemic in majority of these West African countries was the lack of local expertise [22]. According to Christian Bréchot, the president of the Pasteur Institute in Paris, *"the Ebola virus outbreak exposed the shortage of skilled scientists and health-care workers able to diagnose the disease"* [23]. Similarly, Ameenah Gurib-Fakim, a scientist and the president of Mauritius, wrote that *"the shortage of clinical scientists, epidemiologists and diagnostics laboratories to survey and curtail the disease played a major role in the devastating impact witnessed during the Ebola virus crisis"* [24]. Furthermore, Ameenah Gurib-Fakim stated that *"the Ebola virus crisis highlighted the grave disconnect between policy and research and it revealed the absence of strong and credible institutions and underdeveloped medical research systems in Africa"* [24]. Although the immediate crisis is over and Ebola has vanished from the headlines [25], it would be a colossal error to let down our guard and declare that the war is over, as indeed most countries in Africa still remain vulnerable to a resurgence of Ebola or other forms of communicable disease outbreaks [25]. It is high time African governments started investing in the development of physician-scientists. Without their strong commitment to these endeavours, the transition of basic science research to clinical practice i.e. bench to bedside [26] would be significantly compromised [27].

### Factors affecting training of physician-scientist: the peculiar case of Africa

**High cost of biomedical research laboratory set-up**: Due to prohibitive costs of biomedical infrastructure and laboratory set-up, many medical graduates were not exposed to state-of-the-art practical and pedagogical innovations in the field of biomedical sciences [5].

**Dearth of interest in biomedical research by graduates**: The morale to pursue a career in biomedical research by most medical graduate in the African setting is low. This was shown in a study by Azu et al (2013), of the 167 first-year undergraduate medical students who completed a questionnaire on their choice of career post medical school, only 27.6% were interested in academic career in basic medical sciences. This dearth of interest in basic sciences recorded by the participants was ascribed to lack of interest in research as indicated by 48% of the participants, not clinically-orientated (20%), while 12.3% found a career in basic medical sciences an unattractive choice [28].

**Poor remuneration for physician-scientist**: Physicians who choose the biomedical research pathway are poorly remunerated in comparison to their clinician counterpart [29]. This disparity in remuneration and seemingly poor training infrastructure makes the choice of becoming a physician-scientist less attractive in the African clime. These factors militate against training clinician scientists at the postgraduate level; and medical graduates tend to opt for the more financially viable and faster option. Individuals who are determined to pursue this career path often tend to go in search of such opportunities further afield.

**Limited funds dedicated to biomedical research**: An important benefit of training physician-scientists is their dedication towards preventive (vaccine discovery) and improved therapeutic (drug discovery) management of diseases [30]. However, funding is major factor in sustaining a physician scientist career [29, 30]. Though bearing about 90% of global disease burden, Africa access only 10% of globally available health research funding [31]. Similarly, governmental funding for doctors who wish to follow a research path are very sparse and most clinicians are either not experienced in grant writing or are unwilling to pass through the rigor of securing research grants. When funding is being trimmed due to austerity measures, there is a higher likelihood of researchers losing their funding [29, 32]; hence the risk of losing such grants may be a deterrent to passionately pursuing a physician-scientist career in Africa. Furthermore, weak intra-Africa networking in the area of funding research has also hampered the effective use of the sparse resources for the production of critical mass of quality scientists, career opportunities and incentives to retain the few available scientists [31].

**Lack of a well-defined clinician researcher career path**: The high burden of diseases in Africa [33-35] demands a home-grown solution, hence the need for a capable physician-scientist workforce to participate/lead clinical translational research to accelerate the potential of scientific breakthroughs in the field of medicine. Unfortunately, there are no support structures established in the undergraduate and postgraduate medical training programmes in most African countries for physicians to transit into physician-scientist career path [5] except for a few emerging programmes being initiated by academic institutions in South Africa [13, 14]. Unlike many training programmes in Western Europe and the Americas, the fact that most medical training institution in Africa lack a well-defined clinician researcher career path in academic medicine is an additional barrier to commencement and continuance of the physician-scientist training programme. To be able to overcome the burden of diseases in Africa, in-depth scientific research needs to be made with regards to the basis of common diseases; and there is nobody better poised to tackle this problem head-long, than the Africa-trained physician-scientist.

**Demand for basic medical services**: Due to the high demand for basic medical services, medical graduates in Africa are required to go into immediate employment as a service provider. Such forces beyond the power of the affected individuals also contribute immensely to the unlikelihood of a medical graduate pursuing a physician-scientist career directly from medical school. The high clinical workload often makes it difficult if not impossible to secure dedicated time to research in a clinical position [30].

**Recommendations**: The interdisciplinarity derived from becoming a physician-scientist comes at a price. Irrespective of which one you are mostly involved in, there is a trade-off of deficiency in the less practiced profession. In times of funding shortage, physician-scientists tend to retreat to the more financially viable discipline i.e. “clinical practice” [32]. Clearly, there exists a dichotomy within and for the soul of an aspiring physician-scientist and governmental and non-governmental agencies should put structures in place for better transition of African medical graduates (who aspire) to pursue the path of a physician-scientist. In Africa, clinical translational research as a component of the undergraduate curriculum has not received the deserved attention in many medical schools [36]. There is therefore an urgent need to re-assess and improve on the existing metrics of research among medical trainees in Africa [37]. It is recommended that all undergraduate medical students be exposed to a reasonable degree of biomedical research. This would help in creating awareness about possible physician-scientist career options. In addition, all postgraduate residency training programmes should incorporate and emphasize extensive research involvement. To further combat the present uphill battle, more hospitals should be supported by the government of various African states, to fund clinicians who require dedicated time to perform basic medical research, in various medical fields. The extended length of time required to finish the basic medical degree, specialize and do a PhD, needs to be shortened to incentivize the “brave” clinicians who are willing to delve into the basic science world. This can be achieved by the establishment of accelerated programmes such as the MD/MBChB-PhD programmes across Africa. Collaborative linkages should also be established across Africa, for countries with weaker physician-scientist training structures to be able to “piggy-back” on the curriculum of the well-established African countries. Overall, the journey to developing a breed of physician-scientists in Africa is still a long one; but we need to leverage on the lessons that we have learnt in the past to improve the present and provide hope for the future of African medical research. It is high-time Africa acted strategically to accelerate generation of talented physician-scientists workforce, create an enabling environment and incentives to support/retain existing physician-scientists and attract back those in diaspora.

## Conclusion

Investing in an innovative, structured programme that ensures the training of new generation of physician-scientists workforce is essential if Africa is to mitigate the numerous disease challenges facing greater proportion of her populace. There is an urgent need for governments in all African countries to support academic and research institutions in providing better research enabling environment, accelerate the generation and retention of quality physician-scientists, create career opportunities in biomedical research and make available incentives for developing effective tools to improve clinical practices. This will enhance capacity development for clinical research within the continent and reduced the overt dependence of clinical practice on research findings from advanced “first world” countries.

## Competing interests

The authors declare no competing interest.
